# William O. Miller

**Published:** 1899-12

**Authors:** 


William O. Miller, of Rochester, a senior in the Medical Department of the University of Buffalo, died Thursday, November 23, 1899, at his boarding house, No. 152 Mariner street, aged 24 years. On the Sunday previous one finger of his right hand became numb and he suffered with pain in his right side. Paralysis gradually spread until Thursday morning, when he died. Drs. Putnam, Phelps and Cary attended Mr. Miller professionally. It is stated that he died of neuritis. The funeral was held from the University at 9.30 o'clock Friday morning, November 24th, and the remains were taken to Rochester.

